# Diethyl­ammonium anilino(meth­oxy)­phosphinate

**DOI:** 10.1107/S1600536808033023

**Published:** 2008-10-22

**Authors:** Zhiyong Fu, Xiaoling Liu

**Affiliations:** a School of Chemistry and Chemical Engineering, South China University of Technology, Guangzhou, People’s Republic of China

## Abstract

The title compound, [Et_2_NH_2_][(EtO)PO_2_(C_6_H_5_NH)] or C_4_H_12_N^+^·C_8_H_11_NO_3_P^−^, is a molecular salt with two anions containing PO_3_N groupings  and two cations in the asymmetric unit. A network of N—H⋯O hydrogen bonds link the cations and anions into a two-dimensional network.

## Related literature

For the use of *N*-substituted phospho­ramidic acids in the synthesis of pyrophosphate groups, see: Quin & Jankowski (1994 ▶). For a corresponding dimer complex with similar P—O and P—N connections, see: Andrianov *et al.* (1977 ▶).
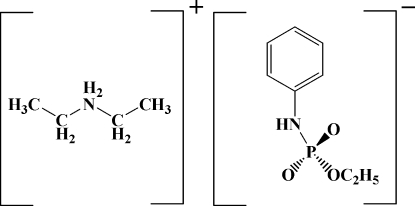


## Experimental

### 

#### Crystal data

C_4_H_12_N^+^·C_8_H_11_NO_3_P^−^
*M* *_r_* = 274.29
Orthorhombic, 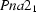

*a* = 14.341 (3) Å
*b* = 12.785 (2) Å
*c* = 15.997 (3) Å
*V* = 2933.0 (9) Å^3^
*Z* = 8
Mo *K*α radiation
μ = 0.19 mm^−1^
*T* = 173 (2) K
0.40 × 0.20 × 0.08 mm

#### Data collection

Bruker SMART CCD diffractometer
Absorption correction: multi-scan (*SADABS*; Sheldrick, 1996 ▶) *T*_min_ = 0.957, *T*_max_ = 0.989
5062 measured reflections
4847 independent reflections
4043 reflections with *I* > 2σ(*I*)
*R*_int_ = 0.017

#### Refinement

*R*[*F* ^2^ > 2σ(*F* ^2^)] = 0.039
*wR*(*F* ^2^) = 0.081
*S* = 1.03
4847 reflections
325 parameters
1 restraint
H-atom parameters constrained
Δρ_max_ = 0.16 e Å^−3^
Δρ_min_ = −0.23 e Å^−3^
Absolute structure: Flack (1983 ▶), 2649 Friedel pairs
Flack parameter: 0.06 (4)

### 

Data collection: *SMART* (Bruker, 1996 ▶); cell refinement: *SAINT* (Bruker, 1996 ▶); data reduction: *SAINT*; program(s) used to solve structure: *SHELXS97* (Sheldrick, 2008 ▶); program(s) used to refine structure: *SHELXL97* (Sheldrick, 2008 ▶); molecular graphics: *SHELXTL* (Sheldrick, 2008 ▶); software used to prepare material for publication: *SHELXTL*.

## Supplementary Material

Crystal structure: contains datablocks global, I. DOI: 10.1107/S1600536808033023/ez2138sup1.cif

Structure factors: contains datablocks I. DOI: 10.1107/S1600536808033023/ez2138Isup2.hkl

Additional supplementary materials:  crystallographic information; 3D view; checkCIF report

## Figures and Tables

**Table 1:** Selected bond lengths (Å)

N1—P1	1.670 (2)
P1—O1	1.4798 (19)
P1—O3	1.4994 (17)
P1—O2	1.5979 (18)
N2—P2	1.651 (2)
P2—O5	1.4897 (18)
P2—O4	1.4983 (19)
P2—O6	1.5969 (17)

**Table 2:** Hydrogen-bond geometry (Å, °)

*D*—H⋯*A*	*D*—H	H⋯*A*	*D*⋯*A*	*D*—H⋯*A*
N1—H1*B*⋯O4	0.86	2.13	2.954 (3)	161
N2—H2*B*⋯O1	0.86	2.04	2.895 (3)	173
N3—H3*C*⋯O4	0.90	1.88	2.742 (3)	160
N4—H4*A*⋯O3	0.90	1.94	2.792 (3)	158
N3—H3*B*⋯O3^i^	0.90	1.89	2.788 (3)	174
N4—H4*B*⋯O5^ii^	0.90	1.74	2.637 (3)	172

Symmetry codes: (i) 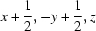; (ii) 
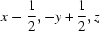
.

**Fig. 1.**
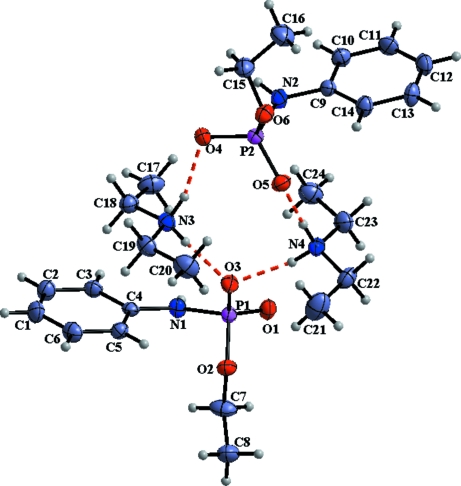
The molecular structure of (I), with atom labels and 50% probability displacement ellipsoids for non-H atoms. The red dashed lines indicate the hydrogen bonds.

**Fig. 2.**
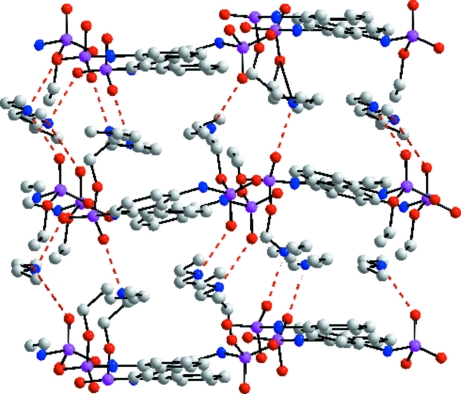
The packing of (I), viewed down the b axis, showing the N—H···O hydrogen bonds between the diethylamine cations and the N-ethoxy phosphoryl-phenylamide anions. H-atoms have been omitted for clarity.

## supplementary crystallographic information

### Comment

N-substituted phosphoramidic acids are useful agents in the synthesis of pyrophosphate groups (Quin & Jankowski,1994). However the structures of these kinds of materials have not been well characterized. By the use of the O,*N*-substituted phosphoramidic acids as ligands in the preparation of new metal phosphate frameworks, we obtained the title compound as a salt of the O,*N*-substituted phosphoramidic acid.

As shown in Fig. 1, the asymmetric unit of the title compound is composed of two diethyl-amine cations, and two *N*-ethoxyphosphoryl-phenylamide anions. The geometrical parameters of the independent anions are similar. The phosphorus atoms have tetrahedral coordination geometries. The shortest P—O bond lengths correspond to the P=O double bonds. The longest P—O distances are due to the influence of the –OEt group (Andrianov *et al.*,1977). The O—P—O and O—P—N bond angles range from 102.961 (98)–119.158 (108)° and 105.126 (113)–112.197 (117)°, indicating that the geometries of the tetrahedra are slightly distorted.

Hydrogen bonds exist between the diethyl-amine cations and the *N*-ethoxyphosphoryl-phenylamide anions. The N—H···O connections result a two dimensional packing motif (Fig. 2).

### Experimental

A solution of aniline (12.5 mmol) and 12.5 mmol of Et_2_NH in 15 ml of ether was added to a solution of 12.5 mmol of ethyl phosphorodichloridate in 15 ml of ether. After 20 h, the solution was filtered and the filtrate was evaporated to give a powder. The powder was dissolved in 30 ml of an acetone-water mixture (1:1) containing 1 g of NaOH. After 10 min, the solvent was evaporated and the residue dried *in vacuo*. Recrystalization of the precipitate from a chloroform solution yielded crystals of the title compound.

### Refinement

H atoms were positioned geometrically and refined using a riding model, with N—H = 0.86–0.90 Å, C—H = 0.93–0.97 Å and with *U*_iso_(H) = 1.2 (1.5 for methyl groups) times *U*_eq_(C). >.

### Crystal data

**Table:**

C_4_H_12_N^+^·C_8_H_11_NO_3_P^−^	*F*(000) = 1184
*M**_r_* = 274.29	*D*_x_ = 1.242 Mg m^−^^3^
Orthorhombic, *P**n**a*2_1_	Mo *K*α radiation, λ = 0.71073 Å
Hall symbol: P2c-2n	Cell parameters from 4847 reflections
*a* = 14.341 (3) Å	θ = 3.3–25.0°
*b* = 12.785 (2) Å	µ = 0.19 mm^−^^1^
*c* = 15.997 (3) Å	*T* = 173 K
*V* = 2933.0 (9) Å^3^	Needle-like, colorless
*Z* = 8	0.4 × 0.2 × 0.08 mm

### Data collection

**Table:**

Bruker SMART CCD diffractometer	4847 independent reflections
Radiation source: fine-focus sealed tube	4043 reflections with *I* > 2σ(*I*)
graphite	*R*_int_ = 0.002
ω scans	θ_max_ = 25.0°, θ_min_ = 3.3°
Absorption correction: multi-scan (SADABS; Sheldrick, 1996)	*h* = −17→17
*T*_min_ = 0.957, *T*_max_ = 0.989	*k* = −15→15
5062 measured reflections	*l* = −19→18

### Refinement

**Table:**

Refinement on *F*^2^	Secondary atom site location: difference Fourier map
Least-squares matrix: full	Hydrogen site location: inferred from neighbouring sites
*R*[*F*^2^ > 2σ(*F*^2^)] = 0.039	H-atom parameters constrained
*wR*(*F*^2^) = 0.081	*w* = 1/[σ^2^(*F*_o_^2^) + (0.0363*P*)^2^ + 0.5497*P*] where *P* = (*F*_o_^2^ + 2*F*_c_^2^)/3
*S* = 1.03	(Δ/σ)_max_ = 0.001
4847 reflections	Δρ_max_ = 0.16 e Å^−^^3^
325 parameters	Δρ_min_ = −0.23 e Å^−^^3^
1 restraint	Absolute structure: Flack (1983), 2649 Friedel pairs
Primary atom site location: structure-invariant direct methods	Flack parameter: 0.06 (4)

### Special details

**Table:**

Geometry. All e.s.d.'s (except the e.s.d. in the dihedral angle between two l.s. planes) are estimated using the full covariance matrix. The cell e.s.d.'s are taken into account individually in the estimation of e.s.d.'s in distances, angles and torsion angles; correlations between e.s.d.'s in cell parameters are only used when they are defined by crystal symmetry. An approximate (isotropic) treatment of cell e.s.d.'s is used for estimating e.s.d.'s involving l.s. planes.
Refinement. Refinement of *F*^2^ against ALL reflections. The weighted *R*-factor *wR* and goodness of fit *S* are based on *F*^2^, conventional *R*-factors *R* are based on *F*, with *F* set to zero for negative *F*^2^. The threshold expression of *F*^2^ > σ(*F*^2^) is used only for calculating *R*-factors(gt) *etc*. and is not relevant to the choice of reflections for refinement. *R*-factors based on *F*^2^ are statistically about twice as large as those based on *F*, and *R*- factors based on ALL data will be even larger.

### Fractional atomic coordinates and isotropic or equivalent isotropic displacement parameters (Å^2^)

**Table:**

	*x*	*y*	*z*	*U*_iso_*/*U*_eq_	
N1	0.50725 (13)	0.16964 (16)	0.52519 (13)	0.0230 (5)	
H1B	0.5374	0.2242	0.5414	0.028*	
N4	0.30305 (14)	−0.01440 (16)	0.74774 (12)	0.0265 (5)	
H4A	0.3436	−0.0013	0.7061	0.032*	
H4B	0.2451	−0.0069	0.7270	0.032*	
C1	0.4316 (2)	0.1694 (2)	0.27143 (19)	0.0359 (7)	
H1A	0.4151	0.1696	0.2152	0.043*	
C2	0.45479 (18)	0.2624 (2)	0.31172 (18)	0.0336 (7)	
H2A	0.4541	0.3251	0.2823	0.040*	
C3	0.47881 (17)	0.2616 (2)	0.39546 (16)	0.0269 (6)	
H3A	0.4937	0.3242	0.4219	0.032*	
C4	0.48107 (16)	0.1682 (2)	0.44095 (16)	0.0220 (6)	
C5	0.45765 (17)	0.0754 (2)	0.39961 (16)	0.0242 (6)	
H5A	0.4583	0.0122	0.4285	0.029*	
C6	0.43349 (18)	0.0772 (2)	0.31564 (18)	0.0315 (7)	
H6A	0.4183	0.0150	0.2888	0.038*	
C7	0.63999 (18)	−0.0209 (2)	0.5543 (2)	0.0407 (7)	
H7A	0.6703	0.0043	0.6046	0.049*	
H7B	0.6556	0.0261	0.5087	0.049*	
C8	0.6729 (2)	−0.1300 (2)	0.53435 (19)	0.0415 (7)	
H8A	0.7393	−0.1297	0.5266	0.062*	
H8B	0.6432	−0.1540	0.4841	0.062*	
H8C	0.6572	−0.1760	0.5797	0.062*	
C21	0.3111 (3)	−0.1992 (2)	0.7038 (2)	0.0540 (9)	
H21A	0.3195	−0.2693	0.7240	0.081*	
H21B	0.3596	−0.1828	0.6645	0.081*	
H21C	0.2516	−0.1935	0.6769	0.081*	
C22	0.3154 (2)	−0.1243 (2)	0.77572 (17)	0.0351 (7)	
H22A	0.2671	−0.1417	0.8157	0.042*	
H22B	0.3752	−0.1312	0.8036	0.042*	
C23	0.3172 (2)	0.0639 (2)	0.81480 (18)	0.0359 (7)	
H23A	0.3785	0.0545	0.8391	0.043*	
H23B	0.2713	0.0529	0.8585	0.043*	
C24	0.3086 (2)	0.1737 (2)	0.7821 (2)	0.0434 (8)	
H24A	0.3185	0.2224	0.8269	0.065*	
H24B	0.2474	0.1838	0.7591	0.065*	
H24C	0.3544	0.1851	0.7393	0.065*	
P1	0.48758 (4)	0.07915 (5)	0.59834 (4)	0.02230 (16)	
O1	0.52914 (12)	0.12003 (13)	0.67646 (11)	0.0297 (4)	
O2	0.54000 (11)	−0.02352 (13)	0.56616 (11)	0.0259 (4)	
O3	0.38708 (11)	0.04682 (12)	0.59813 (12)	0.0272 (4)	
N2	0.55256 (14)	0.33041 (17)	0.73990 (14)	0.0283 (5)	
H2B	0.5502	0.2687	0.7187	0.034*	
N3	0.72854 (14)	0.40960 (16)	0.50343 (13)	0.0267 (5)	
H3B	0.7820	0.4225	0.5310	0.032*	
H3C	0.6826	0.4046	0.5416	0.032*	
C9	0.55185 (17)	0.3353 (2)	0.82782 (16)	0.0233 (6)	
C10	0.55401 (18)	0.2428 (2)	0.87430 (17)	0.0302 (7)	
H10A	0.5560	0.1789	0.8466	0.036*	
C11	0.55331 (19)	0.2445 (2)	0.96026 (18)	0.0343 (7)	
H11A	0.5552	0.1820	0.9899	0.041*	
C12	0.54987 (18)	0.3381 (2)	1.00280 (18)	0.0346 (7)	
H12A	0.5492	0.3391	1.0609	0.042*	
C13	0.5474 (2)	0.4306 (2)	0.95808 (18)	0.0336 (7)	
H13A	0.5451	0.4941	0.9863	0.040*	
C14	0.54840 (19)	0.4294 (2)	0.87124 (17)	0.0298 (7)	
H14A	0.5467	0.4922	0.8419	0.036*	
C15	0.37515 (18)	0.4351 (2)	0.66248 (18)	0.0357 (7)	
H15A	0.3879	0.3613	0.6542	0.043*	
H15B	0.3479	0.4624	0.6115	0.043*	
C16	0.3085 (2)	0.4486 (3)	0.7328 (2)	0.0479 (8)	
H16A	0.2516	0.4121	0.7205	0.072*	
H16B	0.2955	0.5216	0.7404	0.072*	
H16C	0.3353	0.4207	0.7831	0.072*	
C17	0.7673 (2)	0.2234 (2)	0.5187 (2)	0.0412 (7)	
H17A	0.7725	0.1585	0.4889	0.062*	
H17B	0.7220	0.2164	0.5625	0.062*	
H17C	0.8267	0.2413	0.5424	0.062*	
C18	0.73711 (19)	0.3085 (2)	0.45931 (17)	0.0315 (6)	
H18A	0.7824	0.3149	0.4146	0.038*	
H18B	0.6775	0.2900	0.4347	0.038*	
C19	0.70839 (19)	0.4987 (2)	0.44723 (17)	0.0326 (6)	
H19A	0.6487	0.4876	0.4201	0.039*	
H19B	0.7559	0.5023	0.4041	0.039*	
C20	0.7060 (2)	0.6010 (2)	0.4943 (2)	0.0475 (8)	
H20A	0.6928	0.6570	0.4562	0.071*	
H20B	0.7653	0.6128	0.5204	0.071*	
H20C	0.6583	0.5981	0.5364	0.071*	
P2	0.55732 (4)	0.42782 (5)	0.67233 (4)	0.02279 (16)	
O4	0.56495 (11)	0.37700 (13)	0.58821 (11)	0.0282 (4)	
O5	0.62869 (12)	0.50728 (13)	0.69568 (10)	0.0306 (4)	
O6	0.46099 (11)	0.48965 (13)	0.68041 (12)	0.0294 (4)	

### Atomic displacement parameters (Å^2^)

**Table:**

	*U*^11^	*U*^22^	*U*^33^	*U*^12^	*U*^13^	*U*^23^
N1	0.0269 (11)	0.0204 (12)	0.0219 (11)	−0.0046 (9)	−0.0028 (9)	−0.0019 (9)
N4	0.0218 (12)	0.0335 (13)	0.0241 (11)	0.0008 (9)	−0.0012 (9)	0.0045 (10)
C1	0.0436 (17)	0.0395 (18)	0.0247 (15)	0.0050 (14)	−0.0059 (13)	−0.0015 (14)
C2	0.0404 (16)	0.0300 (16)	0.0304 (16)	0.0006 (13)	−0.0034 (12)	0.0025 (14)
C3	0.0305 (14)	0.0226 (15)	0.0276 (16)	0.0018 (12)	0.0013 (11)	−0.0045 (12)
C4	0.0181 (13)	0.0249 (15)	0.0231 (15)	0.0032 (11)	0.0024 (10)	−0.0017 (11)
C5	0.0255 (14)	0.0204 (14)	0.0268 (16)	−0.0006 (11)	−0.0014 (11)	−0.0014 (12)
C6	0.0319 (16)	0.0342 (16)	0.0284 (16)	−0.0013 (12)	−0.0040 (12)	−0.0077 (14)
C7	0.0223 (15)	0.0437 (18)	0.0561 (19)	0.0006 (13)	0.0027 (13)	−0.0137 (15)
C8	0.0409 (17)	0.0400 (18)	0.0435 (18)	0.0134 (14)	0.0066 (14)	−0.0004 (14)
C21	0.073 (2)	0.0354 (18)	0.054 (2)	−0.0014 (17)	−0.0147 (17)	0.0019 (16)
C22	0.0366 (16)	0.0322 (16)	0.0365 (17)	0.0011 (13)	−0.0049 (13)	0.0116 (14)
C23	0.0386 (17)	0.0401 (17)	0.0291 (15)	0.0011 (13)	−0.0048 (13)	−0.0042 (14)
C24	0.0464 (18)	0.0368 (17)	0.0470 (19)	−0.0006 (14)	0.0049 (15)	−0.0052 (14)
P1	0.0261 (4)	0.0211 (3)	0.0197 (3)	0.0005 (3)	−0.0011 (3)	0.0006 (3)
O1	0.0411 (11)	0.0250 (9)	0.0230 (10)	0.0006 (8)	−0.0046 (9)	0.0006 (9)
O2	0.0239 (10)	0.0239 (10)	0.0298 (11)	0.0019 (7)	0.0000 (7)	−0.0027 (8)
O3	0.0225 (9)	0.0320 (10)	0.0272 (10)	−0.0021 (7)	0.0011 (9)	0.0028 (9)
N2	0.0400 (13)	0.0201 (12)	0.0247 (13)	−0.0001 (10)	0.0015 (10)	−0.0041 (9)
N3	0.0250 (12)	0.0307 (13)	0.0245 (12)	0.0010 (9)	−0.0009 (9)	0.0021 (10)
C9	0.0230 (14)	0.0255 (15)	0.0213 (15)	−0.0023 (11)	−0.0003 (11)	−0.0002 (12)
C10	0.0362 (16)	0.0238 (15)	0.0306 (17)	−0.0010 (12)	0.0000 (12)	−0.0010 (13)
C11	0.0428 (18)	0.0298 (16)	0.0304 (17)	−0.0056 (14)	−0.0008 (12)	0.0090 (13)
C12	0.0383 (17)	0.0454 (19)	0.0202 (16)	−0.0005 (13)	−0.0033 (12)	0.0007 (13)
C13	0.0422 (18)	0.0308 (17)	0.0279 (16)	0.0054 (13)	−0.0027 (12)	−0.0089 (13)
C14	0.0374 (16)	0.0256 (15)	0.0264 (16)	0.0020 (12)	−0.0050 (12)	0.0008 (12)
C15	0.0281 (14)	0.0456 (17)	0.0334 (16)	−0.0015 (13)	−0.0007 (14)	−0.0054 (14)
C16	0.0383 (18)	0.060 (2)	0.045 (2)	−0.0006 (16)	0.0093 (15)	−0.0028 (16)
C17	0.0384 (16)	0.0291 (16)	0.056 (2)	−0.0014 (13)	0.0010 (14)	0.0036 (14)
C18	0.0276 (15)	0.0327 (16)	0.0341 (16)	−0.0017 (12)	0.0008 (11)	−0.0017 (13)
C19	0.0342 (16)	0.0357 (17)	0.0277 (14)	0.0065 (13)	0.0007 (11)	0.0078 (13)
C20	0.054 (2)	0.0384 (18)	0.050 (2)	0.0042 (15)	0.0052 (16)	0.0090 (15)
P2	0.0246 (3)	0.0225 (3)	0.0213 (3)	−0.0017 (3)	0.0002 (3)	−0.0004 (3)
O4	0.0334 (10)	0.0304 (10)	0.0208 (10)	−0.0043 (8)	0.0031 (8)	−0.0011 (9)
O5	0.0292 (10)	0.0307 (10)	0.0320 (11)	−0.0062 (8)	−0.0044 (8)	0.0011 (8)
O6	0.0264 (9)	0.0299 (10)	0.0319 (10)	0.0012 (8)	−0.0018 (8)	−0.0045 (9)

### Geometric parameters (Å, °)

**Table:**

N1—C4	1.399 (3)	N2—C9	1.408 (3)
N1—P1	1.670 (2)	N2—P2	1.651 (2)
N1—H1B	0.8600	N2—H2B	0.8600
N4—C23	1.481 (3)	N3—C18	1.478 (3)
N4—C22	1.485 (3)	N3—C19	1.480 (3)
N4—H4A	0.9000	N3—H3B	0.9000
N4—H4B	0.9000	N3—H3C	0.9000
C1—C6	1.374 (4)	C9—C14	1.391 (4)
C1—C2	1.393 (4)	C9—C10	1.397 (4)
C1—H1A	0.9300	C10—C11	1.375 (4)
C2—C3	1.383 (4)	C10—H10A	0.9300
C2—H2A	0.9300	C11—C12	1.378 (4)
C3—C4	1.399 (4)	C11—H11A	0.9300
C3—H3A	0.9300	C12—C13	1.382 (4)
C4—C5	1.399 (3)	C12—H12A	0.9300
C5—C6	1.387 (4)	C13—C14	1.389 (4)
C5—H5A	0.9300	C13—H13A	0.9300
C6—H6A	0.9300	C14—H14A	0.9300
C7—O2	1.447 (3)	C15—O6	1.444 (3)
C7—C8	1.507 (4)	C15—C16	1.487 (4)
C7—H7A	0.9700	C15—H15A	0.9700
C7—H7B	0.9700	C15—H15B	0.9700
C8—H8A	0.9600	C16—H16A	0.9600
C8—H8B	0.9600	C16—H16B	0.9600
C8—H8C	0.9600	C16—H16C	0.9600
C21—C22	1.498 (4)	C17—C18	1.507 (4)
C21—H21A	0.9600	C17—H17A	0.9600
C21—H21B	0.9600	C17—H17B	0.9600
C21—H21C	0.9600	C17—H17C	0.9600
C22—H22A	0.9700	C18—H18A	0.9700
C22—H22B	0.9700	C18—H18B	0.9700
C23—C24	1.504 (4)	C19—C20	1.509 (4)
C23—H23A	0.9700	C19—H19A	0.9700
C23—H23B	0.9700	C19—H19B	0.9700
C24—H24A	0.9600	C20—H20A	0.9600
C24—H24B	0.9600	C20—H20B	0.9600
C24—H24C	0.9600	C20—H20C	0.9600
P1—O1	1.4798 (19)	P2—O5	1.4897 (18)
P1—O3	1.4994 (17)	P2—O4	1.4983 (19)
P1—O2	1.5979 (18)	P2—O6	1.5969 (17)
			
C4—N1—P1	128.36 (18)	C9—N2—P2	128.40 (19)
C4—N1—H1B	115.8	C9—N2—H2B	115.8
P1—N1—H1B	115.8	P2—N2—H2B	115.8
C23—N4—C22	113.87 (19)	C18—N3—C19	113.6 (2)
C23—N4—H4A	108.8	C18—N3—H3B	108.9
C22—N4—H4A	108.8	C19—N3—H3B	108.9
C23—N4—H4B	108.8	C18—N3—H3C	108.9
C22—N4—H4B	108.8	C19—N3—H3C	108.9
H4A—N4—H4B	107.7	H3B—N3—H3C	107.7
C6—C1—C2	119.3 (3)	C14—C9—C10	117.9 (2)
C6—C1—H1A	120.4	C14—C9—N2	122.5 (2)
C2—C1—H1A	120.4	C10—C9—N2	119.6 (2)
C3—C2—C1	120.1 (3)	C11—C10—C9	121.2 (3)
C3—C2—H2A	120.0	C11—C10—H10A	119.4
C1—C2—H2A	120.0	C9—C10—H10A	119.4
C2—C3—C4	121.1 (3)	C10—C11—C12	120.6 (3)
C2—C3—H3A	119.5	C10—C11—H11A	119.7
C4—C3—H3A	119.5	C12—C11—H11A	119.7
C3—C4—N1	119.7 (2)	C11—C12—C13	119.2 (3)
C3—C4—C5	118.2 (2)	C11—C12—H12A	120.4
N1—C4—C5	122.1 (2)	C13—C12—H12A	120.4
C6—C5—C4	120.2 (2)	C12—C13—C14	120.6 (3)
C6—C5—H5A	119.9	C12—C13—H13A	119.7
C4—C5—H5A	119.9	C14—C13—H13A	119.7
C1—C6—C5	121.2 (3)	C13—C14—C9	120.6 (3)
C1—C6—H6A	119.4	C13—C14—H14A	119.7
C5—C6—H6A	119.4	C9—C14—H14A	119.7
O2—C7—C8	108.4 (2)	O6—C15—C16	110.0 (2)
O2—C7—H7A	110.0	O6—C15—H15A	109.7
C8—C7—H7A	110.0	C16—C15—H15A	109.7
O2—C7—H7B	110.0	O6—C15—H15B	109.7
C8—C7—H7B	110.0	C16—C15—H15B	109.7
H7A—C7—H7B	108.4	H15A—C15—H15B	108.2
C7—C8—H8A	109.5	C15—C16—H16A	109.5
C7—C8—H8B	109.5	C15—C16—H16B	109.5
H8A—C8—H8B	109.5	H16A—C16—H16B	109.5
C7—C8—H8C	109.5	C15—C16—H16C	109.5
H8A—C8—H8C	109.5	H16A—C16—H16C	109.5
H8B—C8—H8C	109.5	H16B—C16—H16C	109.5
C22—C21—H21A	109.5	C18—C17—H17A	109.5
C22—C21—H21B	109.5	C18—C17—H17B	109.5
H21A—C21—H21B	109.5	H17A—C17—H17B	109.5
C22—C21—H21C	109.5	C18—C17—H17C	109.5
H21A—C21—H21C	109.5	H17A—C17—H17C	109.5
H21B—C21—H21C	109.5	H17B—C17—H17C	109.5
N4—C22—C21	111.6 (2)	N3—C18—C17	110.7 (2)
N4—C22—H22A	109.3	N3—C18—H18A	109.5
C21—C22—H22A	109.3	C17—C18—H18A	109.5
N4—C22—H22B	109.3	N3—C18—H18B	109.5
C21—C22—H22B	109.3	C17—C18—H18B	109.5
H22A—C22—H22B	108.0	H18A—C18—H18B	108.1
N4—C23—C24	111.6 (2)	N3—C19—C20	111.6 (2)
N4—C23—H23A	109.3	N3—C19—H19A	109.3
C24—C23—H23A	109.3	C20—C19—H19A	109.3
N4—C23—H23B	109.3	N3—C19—H19B	109.3
C24—C23—H23B	109.3	C20—C19—H19B	109.3
H23A—C23—H23B	108.0	H19A—C19—H19B	108.0
C23—C24—H24A	109.5	C19—C20—H20A	109.5
C23—C24—H24B	109.5	C19—C20—H20B	109.5
H24A—C24—H24B	109.5	H20A—C20—H20B	109.5
C23—C24—H24C	109.5	C19—C20—H20C	109.5
H24A—C24—H24C	109.5	H20A—C20—H20C	109.5
H24B—C24—H24C	109.5	H20B—C20—H20C	109.5
O1—P1—O3	119.11 (11)	O5—P2—O4	118.09 (10)
O1—P1—O2	111.87 (10)	O5—P2—O6	103.68 (10)
O3—P1—O2	103.01 (9)	O4—P2—O6	110.52 (10)
O1—P1—N1	106.21 (10)	O5—P2—N2	112.25 (11)
O3—P1—N1	110.60 (10)	O4—P2—N2	105.31 (11)
O2—P1—N1	105.30 (10)	O6—P2—N2	106.54 (10)
C7—O2—P1	119.30 (16)	C15—O6—P2	118.84 (16)

### Hydrogen-bond geometry (Å, °)

**Table:**

*D*—H···*A*	*D*—H	H···*A*	*D*···*A*	*D*—H···*A*
N1—H1B···O4	0.86	2.13	2.954 (3)	161
N2—H2B···O1	0.86	2.04	2.895 (3)	173
N3—H3C···O4	0.90	1.88	2.742 (3)	160
N4—H4A···O3	0.90	1.94	2.792 (3)	158
N3—H3B···O3^i^	0.90	1.89	2.788 (3)	174
N4—H4B···O5^ii^	0.90	1.74	2.637 (3)	172

Symmetry codes: (i) *x*+1/2, −*y*+1/2, *z*; (ii) *x*−1/2, −*y*+1/2, *z*.

## References

[bb1] Andrianov, V. G., Kalinin, A. E. & Struchkov, Yu. T. (1977). *Zh. Strukt. Khim.* **18**, 310–317.

[bb2] Bruker (1996). *SMART* and *SAINT* Bruker AXS Inc., Madison, Wisconsin, USA.

[bb3] Flack, H. D. (1983). *Acta Cryst.* A**39**, 876–881.

[bb4] Quin, L. D. & Jankowski, S. (1994). *J. Org. Chem.* **59**, 4402–4409.

[bb5] Sheldrick, G. M. (1996). *SADABS* University of Göttingen, Germany.

[bb6] Sheldrick, G. M. (2008). *Acta Cryst.* A**64**, 112–122. 10.1107/S0108767307043930 18156677

